# Targeted Therapies in Psoriatic Arthritis—An Update

**DOI:** 10.3390/ijms24076384

**Published:** 2023-03-28

**Authors:** Sonia Sundanum, Carl Orr, Douglas Veale

**Affiliations:** Centre for Arthritis and Rheumatic Diseases, Dublin Academic Medical Centre, University College Dublin, D04 V1W8 Dublin, Ireland

**Keywords:** psoriatic arthritis, targeted therapy, IL-17 pathway

## Abstract

Psoriatic arthritis (PsA) is a systemic inflammatory condition characterised by multiple clinical manifestations. Over the last decade, significant progress has been made in understanding the pathobiology of the disease. An expanded set of targeted therapies have emerged and have shown efficacy in PsA. Nevertheless, there is still a substantial subset of patients who experience no response or only a partial response to currently licensed therapies. The heterogeneous nature of the disease, together with a varying level of severity at presentation and disease activity during follow-up, brings tremendous challenges to devising management strategies. While there are certain pathophysiological similarities between PsA and rheumatoid arthritis (RA), it has become clear that there are discriminating features between these two conditions at the clinical, cellular, and molecular levels. However, there is a degree of overlap in the clinical approach when treating both PsA and RA, given that many biological and targeted therapies have proven efficacy for both pathologies. With an increasing understanding of the relevance of the IL-23/IL-17 axis in PsA, pharmacological agents blocking this pathway have provided promising possibilities for patients with PsA.

## 1. Introduction

Psoriatic arthritis (PsA) is a heterogeneous, chronic inflammatory condition affecting one in five individuals with psoriasis and has been shown to affect about 0.1% of the population [1,2]. It is characterised by a diverse array of clinical features, which include peripheral arthritis, axial spondyloarthritis, dactylitis, enthesitis, uveitis, psoriasis, and nail disease [3]. PsA is recognised as part of the spectrum of psoriatic diseases [4]. It was once thought to be a relatively benign disease; however, it is now well recognised to have a functional burden similar to other inflammatory arthritic conditions such as rheumatoid arthritis (RA) and the axial spondyloarthritides (axSpA) [5].

PsA is progressive and destructive in a substantial subset of individuals. It has been confirmed that damage occurs early in the disease, with nearly 50% of patients showing evidence of radiographic damage at a median interval disease duration of 2 years [6]. Another cross-sectional study illustrated that a diagnostic delay of 6 months contributed to worse outcomes, including the development of peripheral joint erosions, arthritis mutilans, and poor quality of life [7].

Therefore, there is compelling evidence supporting attempts at early diagnosis and prompt institution of proven disease-modifying therapy in PsA. Despite this awareness, delay in diagnosis remains a major challenge in the care of those with PsA. A population-based survey showed an average diagnostic delay of 5 years from the commencement of musculoskeletal symptoms to a final diagnosis of PsA [8]. Numerous factors likely contribute to the challenge of early identification of PsA; these include the heterogeneity of presentation, often only a minimal acute phase response, and limited objective joint swelling compared with RA. Potentially most importantly, the lack of biomarkers to assist in ascertaining a diagnosis remains challenging [9].

The quest to find an optimal and effective therapy that will induce cure or remission has expanded the research agenda to examine new compounds targeting different pathways involved in the persistence of the signs and symptoms of PsA. Table 1 summarises the targeted therapies discussed in this review with their corresponding mode of action.

## 2. Immunopathology of PsA, Genetics, and Environment

### 2.1. Immunopathology

The importance of T lymphocytes has been consistently shown in psoriasis and PsA [10,11]. The key role of T cells in the immunopathogenesis of PsA is illustrated by the presence of T cell response cytokines, namely interferon γ, IL-2, IL-4 TNFα, and interleukin (IL)-17A, and in both the synovial fluid and inflamed synovium [12]. Over the last decade, studies have highlighted key drivers of disease in PsA, and the focus has shifted significantly, from TNFα and Th1 response cytokines, to Th17 cells, IL-23, and IL-17 [13]. These targets have proven relevant in randomised controlled trials (RCT) and have been approved by international regulatory authorities.

When an antigen is presented to the initial T cell, IL-12 is upregulated. This results in the differentiation and proliferation of Th1 cells contributing to pro-inflammatory cytokines, including TNFα, being released [14]. On the other hand, IL-23 is thought to trigger Th17 cell differentiation [15]. This results in the release of IL-22 and IL-17, leading to the upregulation of TNFα [16].

Abundant B lymphocyte aggregates have been noted in the synovium of patients with PsA. However, it is unclear what role, if any, B-cells have in disease onset and perpetuation. Notably, PsA has not been linked with circulating autoantibodies [17]. In addition, monoclonal antibodies targeting CD20 expressed on B cells have only shown a modest benefit in treating PsA [18].

The synovial tissue in PsA shares similarities to those observed in other spondyloarthritis (SpA) but contrasts with the synovium in RA. Compared to RA, synovial macroscopic examination in PsA reveals a distinctly different vascular pattern with potentially important pathogenic implications [19]. The vascular patterns described in the joints in PsA have been reported to be comparable to those noted in the dermis of the skin and nailfold capillaries in psoriasis, thus suggesting a shared link at the tissue level [20].

In another model, McGonagle and colleagues propose that entheseal structures, which are intimately associated with the synovium, are the initial sites of musculoskeletal disease in PsA [21]. In this model, it is hypothesised that synovitis is triggered by the release of proinflammatory cytokines from the closely related enthesis, an anatomic region at the junction of tendon to bone. In a mouse model, IL-23 resulted in entheseal-predominant inflammatory arthritis comparable to what is seen in SpA with bone erosion and new bone formation [22].

### 2.2. Genetics

PsA has a highly heritable component, especially when compared with RA. The recurrence risk ratio in first-degree relatives compared with the general population is in excess of 27, which is considerably higher than the recurrence risk ratio for psoriasis or RA [23]. Susceptibility to PsA and psoriasis is associated with class I major histocompatibility complex (MHC) alleles. This is contrary to RA, which is linked to class II MHC alleles. Nuances in the specific associated genetic susceptibility to the ‘psoriatic spectrum’ exist. For example, a cross-sectional study demonstrated that HLA-C*06 confers susceptibility for psoriasis but not for PsA. Furthermore, HLA-B*08, B*27, B*38, and B*39 with particular subtypes of these alleles are associated with particular ‘sub-phenotypes’, with symmetric or asymmetric sacroiliitis, enthesitis, dactylitis, and synovitis [24]. Despite these well-characterised genetic associations with sub-phenotypes, no role for genotyping has been established in selecting treatment for individual patients.

### 2.3. Environment

The role of environmental factors as a trigger of PsA in genetically susceptible individuals is the subject of intense research. Environmental risk factors such as trauma (Koebner’s phenomenon), infections, smoking, and immunological triggers (such as rubella vaccination) have each been posited to be risk factors for the onset of PsA [25].

## 3. Unmet Needs in Therapeutics—The Challenges

New treatment paradigms have recently emerged with the recognition of novel therapies targeting specific cytokines and signalling pathways. Despite these expanding therapeutic targets, the overall improvement in response to therapies has been disappointing.

At least 40% of PsA patients exhibit no response or only a partial response. Clinical trials have failed to consistently show an American College of Rheumatology criteria for 20% improvement (ACR20) in more than 60% of patients [3,26]. Therefore, there is growing interest in developing and testing other therapeutic concepts to expand the repertoire of treatment modalities available to treat patients with PsA.

The heterogeneity of PsA challenges practicing rheumatologists in choosing the most suitable therapy for their patients. To address these challenges, international bodies including the Group for Research and Assessment of Psoriasis and Psoriatic Arthritis (GRAPPA) and the European League Against Rheumatism (EULAR) have issued recommendations to guide clinicians [27]. The recent GRAPPA recommendations, published in 2021, suggest addressing all active disease domains and PsA-relevant comorbidities in an individual patient and driving treatment decisions based on the domain with the highest level of activity. However, this approach can be insensitive for the many individuals who exhibit persistent disease in several domains [28].

Combining therapies with different modes of action is an appealing area of interest. This strategy has the theoretical potential to induce remission. One novel approach has been the development of a bispecific TNF-inhibitor (TNFi) and IL-17 inhibitor (targeted variable domain immunoglobulin), which acknowledges the proven efficacy of each cytokine in treating PsA. However, this was not superior in clinical trials to adalimumab in PsA or, indeed, RA. Nevertheless, there were no major safety signals in these relatively small studies, inhibiting two cytokines [29]. In a proof-of-principle clinical trial of this bispecific inhibitor in PsA in inadequate methotrexate responders, the ACR50/ACR70 response and the Psoriasis Area and Severity Index criteria for 75% improvement (PASI75)/PASI90 was statistically superior to adalimumab at multiple time intervals. These promising efficacy results were combined amidst comparable safety parameters to that of the TNFi comparator (adalimumab). However, further development and testing of the agent have not been pursued [30].

Numerous case reports have described improvement in disease manifestations using dual biologic combinations, including TNF-inhibitors (TNFi) and gulselkumab (IL-23 inhibition) or ustekinumab (IL 12-23 inhibition). Given the lack of blinding in these studies, the results must be interpreted cautiously because of the possibility of publication bias [31].

The AFFINITY (**NCT05071664**) multicenter double-blind RCT evaluating a combined strategy of golimumab and guselkumab (IL-23 inhibition) versus guselkumab monotherapy is currently enrolling participants. This study aims to answer important questions about the efficacy and safety of implementing dual cytokine inhibition in the setting of psoriatic arthritis [32].

With an increase in pathogenic understanding of the processes involved in the disease, treatments now include compounds targeting TNF, IL-12/23, IL-23, IL-17, JAK-STAT, and phosphodiesterase type 4 (PDE4). However, a recent systematic review revealed that minimal disease activity (MDA) is only attained in 17% of those prescribed conventional synthetic disease-modifying anti-rheumatic drugs (csDMARD) and in up to only 57% of those on biologic DMARD (bDMARD) [33].

Given the financial costs associated with bDMARDs and tsDMARDs therapy, another important challenge in treating PsA relates to the costs of available treatments. Notwithstanding the reduction in costs of targeted biological therapies in recent years, a more precise selection of initial treatments could be greatly improved if biomarkers, including genetic, cellular, and other molecular markers, were identified and validated in predicting treatment responses. This could conceivably enable patients to experience either minimal disease activity or remission at the earliest stage possible.

## 4. TNFα

TNF inhibitors (TNFi) were the first licensed biologics for the management of PsA and are now commonly utilised. There are five TNFi agents approved for use (adalimumab, etanercept, infliximab, certolizumab pegol, and golimumab). There is good evidence that TNFi can slow or prevent the radiographic progression in peripheral PsA as demonstrated by stable measures of structural damage such as radiological measurements of erosions, and joint space narrowing [34,35,36,37,38]. One of the disappointing aspects of the novel therapies has been the inability to retard a cardinal characteristic of PsA progression, new bone formation.

It is worth noting that the response to TNFi whether used as monotherapy or with concomitant csDMARD (usually methotrexate) does not differ greatly [39]. However, these studies were not appropriately powered to allow a sound conclusion on whether combination therapy provides additional benefits as opposed to TNFi or csDMARD monotherapy in the early stages of PsA.

The SEAM-PsA study evaluated the efficacy of methotrexate monotherapy, etanercept monotherapy or methotrexate in conjunction with etanercept in patients with a relatively short disease duration [39]. One very important finding of this trial was that methotrexate treatment on its own does provide benefits in the management of treatment-naïve patients, which, while an established therapy, has had very little data to date. However, the trial did not include a placebo control arm, making this finding difficult to confirm definitively. Another important finding from this study is that combination therapy confers very little additional benefit over etanercept monotherapy.

TNFi has been demonstrated to be a potent therapy for all disease phenotypes and is recommended by GRAPPA and EULAR for entheseal involvement and dactylitis [27,28]. However, all the key phase III RCTs assessing TNFi in PsA used different measures to evaluate enthesitis, thus rendering comparison challenging [34,35,36,37,38].

With a paucity of data regarding the effectiveness of TNF inhibition on axial disease in PsA, the recommendations stem from data for axSpA studies [40,41,42,43,44]. TNFi appears to have an impact on axial inflammation by reducing pain, decreasing markers of active disease such as C-reactive protein (CRP) and improving the lumbar spinal range of motion. However, it is not entirely clear whether improvement in these indices translates to a true impact on the radiographic progression in the axial skeleton, and, therefore, whether these agents can be considered truly disease-modifying, especially for axial disease [45].

As in RA, uncontrolled inflammation can lead to bone and cartilage destruction and erosion in patients with PsA. One fundamental difference to RA, referenced already, is that the bony architectural changes in PsA are characterised by the presence of both catabolic and anabolic bone changes [46]. These changes suggest the presence of increased resorption (resulting in bone erosions and osteolysis) coupled with enhanced bone formation, such as syndesmophytes and enthesophytes. In PsA, normal bone remodelling homeostasis and the crosstalk between osteoclasts and osteoblasts are disrupted. A number of cytokines and growth factors normally associated with osteoclasts and osteoblasts differentiation are dysregulated, with the result being the complex bone phenotype involving both erosive changes and new bone formation seen in PsA [47]. While TNFi retard the progression of bone erosion, they do not appear to prevent the phenomenon of new bone formation seen in PsA.

**Figure 1:**
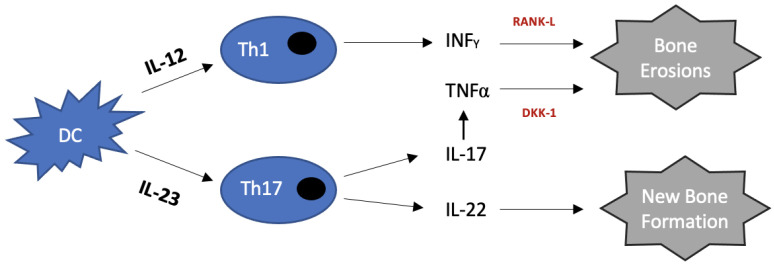
Pathogenesis of psoriatic arthritis. (Adapted with permission from Mantravadi, S. et al., Tumor necrosis factor inhibitors in psoriatic arthritis; published by *Expert Review of Clinical Pharmacology*, 2017. Copyright 2017 by Taylor and Francis) Dendritic (DC) and T helper (Th17 and Th1) cells produce several cytokines. TNFα promotes osteoclastogenesis via RANK-L and inhibits osteoblastogenesis via Dkk-1. This ultimately results in bone erosion formation [49]. IL-22 is involved in new bone formation [50,51].

TNFα plays an important part in the formation of erosions as it induces osteoclast differentiation by triggering the expression of receptor activator nuclear factor-_k_B ligand in the synovium, which is a key player for osteoclast differentiation [48]. TNFα also stimulates the expression of Dickkopf-related protein 1 (Dkk-1) by synovial fibroblasts, which suppress osteoblast differentiation, further promoting the formation of erosions (Figure 1) [48,49]. Based on this knowledge, it is clear that inhibition of TNFα by the currently approved compounds (TNFi) does not prevent new bone formation in PsA. Indeed these findings imply that other cytokines, such as IL-22, may be at the centre of this excessive bone formation [50].

## 5. IL-12/23

Arguably the most important advance over the last two decades has been the recognition that the IL12/23 and IL-17 cytokine axis plays a major role in the biology of psoriatic disease. As a result, growing interest has emerged in developing therapeutic agents targeting this pathway directly via IL-17 inhibition or upstream via inhibition of IL-12 and 23.

Ustekinumab is a human monoclonal antibody targeting the p40 subunit shared by IL-12 and IL-23 [52]. It was first approved for the management of plaque psoriasis following two phase III trials (PHOENIX 1 and 2) [53,54].

Following the PHOENIX trials in psoriasis patients, the PSUMMIT 1 and PSUMMIT 2 Phase III trials were performed to assess ustekinumab in patients with PsA [55,56]. Based on the results of these two trials, ustekinumab received approval from both the Food and Drug Administration (FDA) and naïve European Medicines Agency (EMA) for the management of PsA, representing the first approval of a novel agent for PsA in a decade.

In PSUMMIT 1, 615 TNFi-naïve PsA patients were randomised 1:1:1 to receive a placebo vs. 45 mg vs. 90 mg of ustekinumab. ACR20/50/70 and PASI75 responses were evaluated at 24 weeks, with ACR20 as the primary outcome. Ustekinumab showed superiority to the placebo; in the 90 mg ustekinumab group, the ACR20/50/70 response was achieved in 49.5%, 27.9%, and 14.2%, whereas the placebo responses were 22.8%, 8.7%, and 2.4%, respectively. In PSUMMIT 1, ACR20 response rates were sustained at 52 weeks regardless of concomitant methotrexate use [55].

In contrast to PSUMMIT 1, PSUMMIT 2 included 58% of patients who had previous TNFi exposure but experienced an inadequate response. A clinical response to ustekinumab was shown regardless of prior TNFi exposure; however, the magnitude of the clinical response was lesser in the TNFi-inadequate responders in contrast to the naïve patients [56].

Most of the participants in the TNFi-experienced arm discontinued TNFi due to inadequate response, with most having been treated with at least two prior TNFi. It is possible that this specific group of patients had a more recalcitrant disease phenotype [57].

The IL23–Th17–IL–17 axis has been associated with the activation of osteoclastogenesis and thus promotes erosive changes [58]. Radiographic progression was evaluated in the PSUMMIT 1 and 2 trials using the PsA-modified Van Der Heijde-Sharp (vdH-S) scoring system, which scores erosions and joint space narrowing. At week 24, more participants in the combined ustekinumab-treated group demonstrated no progression compared to the control (91.7% vs. 83.8%, *p* = 0.005). A reduction in progression was also noted in patients originally receiving the placebo who transitioned over to the active arm. The proportion of patients with features of ‘arthritis mutilans’ (‘pencil-in-cup’ or gross osteolysis) also remained at a low level and stable [52]. It is worth noting that, once again, the scoring system employed to evaluate the X-rays of the hands and feet of the patients did not take full account of new bone formation [59]. Furthermore, the duration of the trial may have been too short to assess this aspect of disease modification meaningfully.

Although preliminary efficacy has been demonstrated in an open-label, proof-of-concept trial evaluating ustekinumab in axSpA [60], a large phase III study in axSpA was terminated as the study’s primary and secondary endpoints were not met, thereby raising important questions about the disease-modifying effect of ustekinumab in axial PsA.

## 6. IL-23

Responses in collagen-induced arthritis animal models have demonstrated that loosing the IL-23 gene (p19^−/−^) was protective, whereas a loss of the IL-12 gene (p35^−/−^) worsened arthritis. This led to efforts to inhibit exclusively IL-23 in humans with PsA, which have been shown to be efficacious [61].

IL-23 dysregulation plays a central role in enthesitis. IL-23 expression has been shown to induce enthesitis in mice along with several features which are also seen in humans, including periostitis, entheseal and periosteal new bone formation, and bone erosion. Later, a ‘secondary’ synovitis appears to develop, which has destructive properties similar to ‘arthritis mutilans’ as seen in PsA [22,62].

Guselkumab and risankizumab are monoclonal antibodies targeting the p19 subunit of IL-23, thus, have no biological influence on IL-12.

These therapies have demonstrated clear benefits in psoriasis treatment, with superiority, versus adalimumab and ustekinumab [63,64,65,66]. Guselkumab is the only anti-IL-23p19 currently approved by the EMA and the FDA for the treatment of PsA. Guselkumab 100 mg administered every 4 weeks (Q4W) versus every 8 weeks (Q8W) were studied in the placebo-controlled randomised DISCOVER-1 and DISCOVER-2 phase III trials in active PsA. In both trials, guselkumab administered at both dose frequencies significantly improved the manifestations of PsA compared to a placebo at 24 weeks [67,68]. Sustained improvements were also noted through 1 year [69,70].

In DISCOVER-1, previous exposure to one or two TNFi was allowed but was limited to ∼30% of the study cohort, while all participants in DISCOVER-2 were biologic-naïve. The primary endpoint (ACR20 at week 24) for DISCOVER-1 (N = 381) was experienced significantly by patients in the guselkumab group every Q4W (58%) and Q8W (52%) than in the placebo group (22%) [67]. DISCOVER-2 included a larger population of PsA patients (N = 739); again, significantly greater proportions of patients receiving guselkumab (at both dose regimens; Q4W and Q8W) reached an ACR20 response at week 24 compared to the placebo group [68]. The pooled safety data through 1 year of the two phase III DISCOVER trials did not identify any new safety signals, guselkumab at both doses was well tolerated by PsA patients, and the safety was consistent with that established in psoriasis patients who received treatment with guselkumab [71].

The efficacy of guselkumab in enthesitis resolution is worth mentioning. Among 1118 randomised patients in both DISCOVER trials who had ≥1 Leeds Enthesitis Index (LEI) site examined, 65% had enthesitis at baseline. Guselkumab Q4W and Q8W were superior to the placebo in enthesitis resolution at week 24 (45% and 50% vs. 29%). Enthesitis resolution rates continued to increase, and by week 52, 58% of patients randomised to guselkumab achieved resolution [72]. Nevertheless, direct comparisons with other available treatments in this domain make this finding difficult to interpret.

The COSMOS trial, which exclusively included TNFi-inadequate responders, demonstrated the superiority of guselkumab Q8W (ACR20 response of 44.4% at week 24) compared to a placebo (ACR20 response of 19.8% at 24 weeks), thus suggesting that guselkumab is an appropriate therapy for PsA patients who are intolerant to or lack response to TNFi [73].

The question of radiographic progression was evaluated only in the DISCOVER-2 trial, and progression was significantly lower in the guselkumab Q4W group than in the placebo group at 24 weeks. As referenced earlier, this is one major challenge in clinical trials where scores of radiographic progression are assessed over a relatively short time period.

The long-term efficacy analysis of patients treated with guselkumab through 2 years as part of the DISCOVER-2 was recently published. Mean changes in the PsA-modified vdH-S score from weeks 52 to week 100 indicated that the low rates of progression seen in guselkumab-treated patients at earlier time points continued through week 100 [74].

The efficacy of guselkumab regarding retarding new bone formation was not assessed, and only long-term follow-up will determine whether IL-23 inhibition can delay or prevent new bone formation.

As in the case of ustekinumab, a phase II proof-of-principle study of risankizumab in ankylosing spondylitis failed to meet the primary endpoint [75]. One posited theory is that IL-23 is involved in the initiation of disease but not in the ‘perpetuation’ of axial inflammation in spondyloarthropathy [76,77]. It is unclear how and if this would apply to axial PsA. Interestingly, a post hoc analysis of DISCOVER-1 and DISCOVER-2 reported that patients with active PsA and imaging-proven sacroiliitis who received guselkumab (regardless of the dose regimen utilised) had greater mean improvements in measures of axial disease activity scores than the patients treated with a placebo [78]. Based on these results, the STAR study, a phase IV RCT of guselkumab in biologic-naïve patients with active axial PsA, is currently recruiting participants. It will be able to determine if guselkumab truly has a disease-modifying effect on the axial skeleton in PsA (**NCT04929210**).

## 7. IL17

IL-17 is an inflammatory cytokine produced by Th17 T cells and other cells, and its presence has been demonstrated in plaque psoriasis and enthesitis [79].

The IL-17 superfamily comprises six ligands (IL-17A to IL-17F) which can bind to five subtypes of receptors (IL-17RA to IL-17RE). IL-17A is the best characterised ligand of the IL-17 family. It can exist as a homodimer or in a heterodimer with IL-17F and signals through the IL-17RA and IL-17RC receptor complexes. Once bound to a receptor, IL-17A upregulates inflammatory gene expression [80,81].

While not present in significant concentrations in RA joint fluid, IL-17A-producing conventional CD8 + T cells have been demonstrated in the joint fluid of patients with active PsA, and the titers correlate with disease activity [82,83]. Il-17A has been implicated as an amplifier of the inflammatory response in the entheses, including other cytokines released by resident mesenchymal cells [22,84,85].

**Figure 2:**
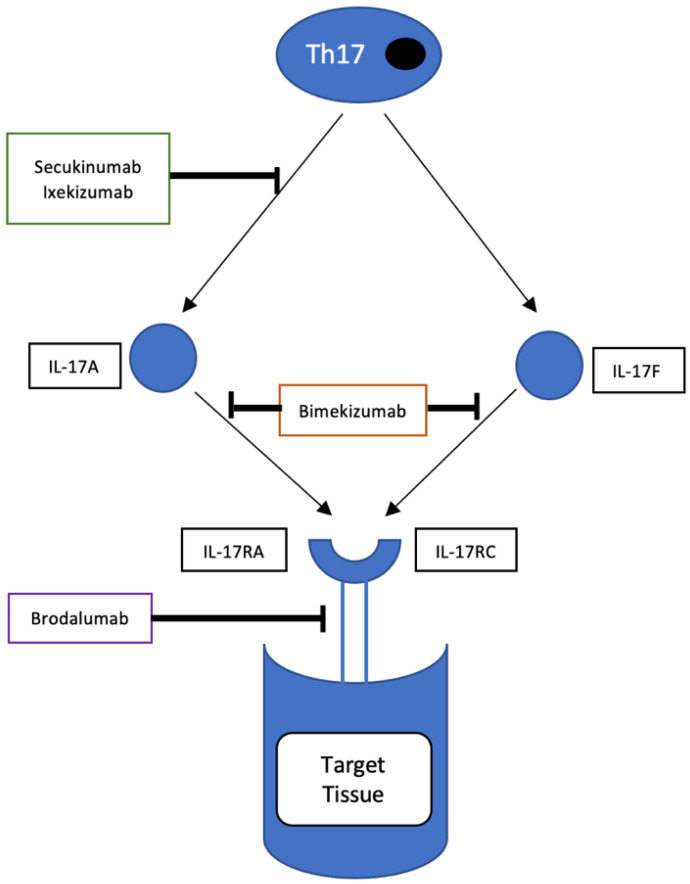
Mechanism of action of IL-17 inhibitors. Secukinumab and ixekizumab bind to IL-17A and inhibit its interaction with the IL-17 receptor. Bimekizumab binds to Il-17A and IL-17F and inhibits the activation of the IL-17RA/RC complex. Brodalumab binds to IL-17RA and blocks the actions of IL-17A,E,F.

With basic science highlighting the importance of the Th17 pathway in PsA, a number of new therapies targeting this pathway have been developed. The mechanism of action of IL-17 inhibitors is shown in Figure 2. Secukinumab is a fully human anti-IL-17A monoclonal antibody approved for the treatment of PsA, psoriasis, and ankylosing spondylitis following the favorable results of several large RCTs [86,87,88,89,90,91].

The results from phase III studies, FUTURE 1 and FUTURE 2, showed that secukinumab results in rapid and significant improvements in the manifestations of PsA, sustained for up to 3 years of treatment. Clinical benefits were noted in biologic-naïve patients and those with prior TNFi treatment [88,89,92,93,94,95,96]. Perhaps not surprisingly, responses were better in those without prior exposure to biologic DMARDs. Only the TNFi non-responders who received the 300 mg dose had a statistically significant benefit compared to the placebo [97]. FUTURE 1 included intravenous loading followed by a subcutaneous regimen and did not assess a dose higher than 150 mg. FUTURE 2 utilised a subcutaneous loading and maintenance dosing of 300 mg, 150 mg, and 75 mg.

FUTURE 1 demonstrated a 50.5% and 50.0% ACR20 response at 24 weeks to secukinumab at 75 mg and 150 mg, respectively, and FUTURE 2 demonstrated a 29.3%, 51.0%, and 54.0% ACR20 response for doses at 75, 150, and 300 mg, respectively, versus 15.3% for the placebo [88,89]. Regulatory bodies subsequently approved the 300 mg and 150 mg dosing regimens for use in PsA.

The data from FUTURE 1 showed that secukinumab significantly inhibited structural damage through week 24 with benefits maintained to 2 years [96]; however, FUTURE 2 did not examine radiographic progression. This efficacy in radiographic disease progression was confirmed in FUTURE 5, which evaluated the effects of secukinumab 300 mg and 150 dose regimens on structural damage through 52 weeks. This study showed that both doses, with or without subcutaneous loading, provided sustained low rates of progression compared to the placebo [98]. IL-17A has been implicated in promoting osteoblast differentiation and thus may stimulate new bone formation [99,100]. A small open-label study of secukinumab demonstrated no worsening of catabolic and anabolic bone changes in PsA patients. This study utilised MRI and power doppler ultrasound to assess structural damage as opposed to conventional radiographs used in most clinical trials. These are promising data showing that secukinumab may have an impact on blocking new bone formation; however, the lack of a placebo control makes it challenging to interpret these results [101].

The safety data from Future 1 and 2 were similar to those in psoriasis. Importantly, there were no new cases of tuberculosis. Most adverse events were due to upper respiratory tract infections, which were only marginally increased in incidence in the secukinumab-treated group; these occurrences did not appear to be dose related. Candida infections were more prevalent in the secukinumab-treated arm [89,96].

Ixekizumab is a high-affinity monoclonal antibody that has specificity for IL-17A. Ixekizumab demonstrated its superiority over a placebo in the SPIRIT-P1 and SPIRIT-P2 phase III trials in patients with active PsA and is approved for use in PsA, psoriasis, and asSpA [102,103].

Secukinumab and ixekizumab have both demonstrated efficacy in outcome measures of axial inflammation and are therapeutic options for patients with axial PsA [104,105].

There has been a welcome increase in head-to-head randomised control trials in PsA in recent publications. The efficacy and safety of ixekizumab were compared with adalimumab; ixekizumab was superior in achieving concurrent improvement of joint and skin disease in PsA patients who experienced an inadequate response to csDMARDs [106]. McInnes et al. evaluated the efficacy and safety of secukinumab with adalimumab; secukinumab was at least as efficacious as the TNFi on musculoskeletal endpoints but exceeded on skin outcomes [107]. These two important studies have challenged the historic precedent for selecting TNFi as the initial biological agent for PsA patients and have highlighted potential alternatives with differing mechanisms of action.

Another agent targeting the IL-17 pathway is brodalumab, a fully monoclonal antibody with a unique mode of action. Brodalumab binds with high affinity to the IL-17 receptor subunit A (IL-17RA), resulting in the blockade of the action of multiple proinflammatory cytokines of the IL-17 superfamily beyond that of IL-17A alone. This agent is approved for the treatment of psoriasis by the FDA and EMA and is approved only in Japan for PsA. The findings from the phase II AMVISION-1 and AMVISION-2 trials suggest that brodalumab is a promising therapy for PsA. Approximately 30% of patients included in these two trials had prior treatment with biologics. Significantly more patients treated with brodalumab (at both doses of 140 mg and 210 mg at week 0 and 1 and every 2 weeks) reached an ACR20 response at 16 weeks compared to placebo (45.8% and 47.9% for the 140 mg and 210 mg doses, respectively, versus 20.9% in the placebo-treated group, *p* < 0.0001). These results were sustained at week 24. ACR50/70 and PASI75/90/100 responses were achieved by significantly greater proportions of patients who received brodalumab and this group also experienced significantly higher proportions of dactylitis and enthesitis resolution versus the placebo group. The adverse event rates in the brodalumab groups were similar to the placebo [108]. This may represent another treatment option for patients with PsA.

IL-17F has the highest structural homology with IL-17A (close to 50%), with overlapping biology with IL-17A. However, the role it plays in immune-mediated inflammatory diseases remains less clear. IL-17A and IL-17F bind to the same complex of IL-17RA and IL-17RC [109]. IL-17F has similar actions to IL-17A but has less potent properties since it has less affinity for the IL-17RA/RC complex. However, levels of IL-17F have been shown to be higher in psoriatic skin tissue and serum (30-fold higher on average) than levels of IL-17A [110]. Studies have established that neutralising both IL-17 ligands (IL-17A and IL-17F) more potently suppresses in-vitro cytokine responses and neutrophil chemotaxis than targeting either ligand alone [111]. This biological basis has informed the development of dual and bispecific IL-17A and IL-17F inhibitors, and the clinical data thus far have been encouraging, especially for bimekizumab. Data from the phase 2b BE ACTIVE study showed that Bimekizumab, a monoclonal IgG1 antibody which selectively inhibits IL-17F and IL-17A, accounted for rapid improvements in skin and joint endpoints in PsA patients [112].

The results of two phase III clinical trials of bimekizumab for PsA have been very recently reported. The BE OPTIMAL trial included only biologic naïve-patients allocated to one of three groups: bimekizumab, a placebo, or adalimumab (the reference group). However, the study was not powered to allow statistical comparisons between adalimumab and bimekizumab. Here, an ACR50 response at 16 weeks was the selected primary endpoint. The study achieved its primary endpoint, with a significantly larger proportion of patients experiencing an ACR50 at 16 weeks in the bimekizumab-treated group (44%) versus the placebo group (10%). Active treatment also inhibited radiographic progression in patients in both the overall population (a subgroup with elevated CRP) and in those with at least one bone erosion at baseline [113].

The BE COMPLETE study included patients with prior TNFi use and included two groups—a bimekizumab-treated group and a placebo-treated group. Bimekizumab was superior to the placebo in achieving an ACR50 response at 16 weeks (43% versus 7%, respectively). Moreover, bimekizumab demonstrated rapid improvements in clinical manifestations of PsA, with separation from the placebo as early as week 4 [114].

The results from these trials highlight another possible agent for PsA. However, some important questions remain, especially regarding the efficacy of bimekizumab in the resolution of dactylitis and enthesitis. BE COMPLETE did not include these domains in the secondary efficacy analyses due to low numbers of patients with dactylitis and enthesitis, resulting in a lack of statistical power to assess those endpoints adequately. As such, these domains were analysed by pooling data from the two trials, and the results were reported by BE OPTIMAL. Bimekizumab only performed modestly better than the placebo for these disease domains. Complete enthesitis resolution was seen in 50% of those treated with bimekizumab compared to 35% in the placebo group at week 16, *p* = 0.0083 and complete dactylitis resolution was observed in 76% in the bimekizumab group compared to 51% in the placebo group, *p* = 0.0022 (an odds ratio of 1.9 for enthesitis resolution; an odds ratio of 3.4 for dactylitis resolution). Disappointingly, dual blockade therapy appears to have similar efficacy in both biologic-naïve patients and those previously treated with TNFi. In fact, the ACR50 response was nearly the same in BE COMPLETE and BE OPTIMAL. While bimekizumab seems to be a new effective, safe therapy for PsA, the true advantage of combined inhibition of IL-17A and IL-17F versus IL-17A alone remains to be demonstrated. Bimekizumab was superior to other therapeutic targets when efficacy was compared in direct head-to-head trials in psoriasis, with higher rates of fungal infections than adalimumab and secukinumab [115,116,117]. It is likely that direct comparative studies of bimekizumab versus other therapies for PsA will provide a better understanding of the future role of this compound in the PsA treatment algorithm.

## 8. Janus Kinase (JAK) Inhibitors

The JAK family of non-receptor, intracellular tyrosine kinases comprises four members: JAK1-3 and tyrosine kinase (TYK) 2. They represent a family of intracellular signal transducers with downstream regulation of activators of transcription (STAT). The JAK-STAT signalling pathway can be activated by many important proinflammatory cytokines involved in the pathogenesis of PsA, such as those related to the IL-12/23 and the IL-17 axes [118,119,120]. Consequently, targeting the JAK-STAT pathway via small molecules, JAK inhibitors (JAKi), has a biological basis and has been the subject of intense research in PsA.

Tofacitinib, an oral JAKi which inhibits signalling through JAK3 and JAK1, is approved for use in PsA. The approval of tofacitinib for PsA was based on two phase III RCTs: OPAL Broaden, which included patients naïve to TNFi and OPAL Beyond, which included patients who had an inadequate response to previous TNFi use. [121,122] OPAL Broaden included adalimumab as an active comparator. In the TNFi-naïve patients, the efficacy of tofacitinib with respect to joint and skin responses was (disappointingly) similar to that of adalimumab (ACR20 response at 3 months was achieved in 50% of the 5 mg-tofacitinib group, 60% of the 10 mg-tofacitinib group, and 52% of the adalimumab group versus 33% in the placebo-treated group) [121]. Nevertheless, and importantly, significant improvements were also seen in the patients who had prior inadequate responses to TNFi [122].

The biology of the JAK-STAT signalling pathway is complex but it is clearly an important modulator in normal cell survival and growth [123]. Consequently, concerns have been articulated about the true selectivity of the small molecular inhibitors, and the safety of their use regarding malignancy risks and serious infections. Four cases of herpes zoster infections were reported in OPAL Broaden and three in OPAL Beyond in patients treated with tofacitinib. While these data confirm a statistically significant increased risk of herpes zoster infection, the increased clinical risk is poorly understood. An open-label long-term extension study, analysed at 3 years, which included patients from the two phase III tofacitinib PsA RCTs did not reveal any new safety signals [124]. However, further long-term safety data will be required, especially with respect to the risk of neoplasms. Furthermore, as a result of the ORAL surveillance study in RA, higher rates of major cardiovascular events and malignancies in tofacitinib-treated patients compared to TNFi-treated patients appear to have been confirmed [125].

Clinical trials assessing the efficacy and safety of various JAKi in PsA have exploded in recent years. Upadacinitib, an oral JAK1 selective inhibitor, was evaluated in a phase III trial that included patients who had an inadequate response to csDMARD [126] and those who had had inadequate responses to TNFi. [127] Upadacitinib was superior to the placebo in these trials and was non-inferior to adalimumab in SELECT-PSA1. Rates of herpes zoster were increased compared to TNFi but were not more serious than infections in those not treated with JAKi [126]. Long-term safety data are awaited regarding upadacinitib, although it is to be noted that it has been approved across the highest number of indications in its class.

Filgotinib is an oral selective JAK1 inhibitor. It showed efficacy over a placebo in a phase II clinical trial, which included patients with PsA who had an inadequate response to csDMARD [128].

TYK2 is an intracellular kinase member of the JAK family and signals through the JAK–STAT pathway. It mediates downstream signalling by numerous cytokines, including IL-23, which are targets in PsA and psoriasis. Unlike the other members of the JAK family (JAK1/2/3), the TYK2 signalling pathways are restricted to select immune pathways [129,130]. Deucravacitinib is a novel oral selective TYK2 inhibitor which binds to the regulatory domain of TYK2, thus rendering the enzyme inactive. Deucravacitinib showed promising results in a phase II trial which included patients who had an inadequate response to csDMARD, and two phase III trials are currently enrolling to further assess the results in PsA patients [131,132,133].

The second generation of JAKi (filgotinib and upadacitinib) were initially developed to increase the selectivity of inhibition while maintaining or improving efficacy. It is hoped that selective inhibition will lead to superior safety endpoints compared to non-selective JAKi, though this has not yet been confirmed in head-to-head studies.

## 9. Other Targets

Menon et al. reported that IL-17-producing T cells (mainly CD8 + T cells) are more abundant in the synovial fluid of those with PsA than RA. The authors also demonstrated that raised synovial fluid IL-17 + CD4-negative T cells correlated with elevated acute phase reactants, features of active power doppler ultrasound synovitis and erosive disease [82]. Abatacept, a selective T cell co-stimulation modulator fused to the extracellular domain of human cytotoxic T-lymphocyte-associated antigen 4 (CTLA-4), was studied as a potential pharmacologic intervention in PsA. However, when assessed in a RCT, abatacept only demonstrated a modest response with marginally better efficacy in patients naïve to TNFi compared to patients who had prior exposure to TNFi [134]. Abatacept is approved for use in PsA, but given its relatively low efficacy, the EULAR recommendations suggest that its use should be considered only after other bDMARD classes have failed [27].

There are other cellular and molecular targets approved for the treatment of RA and that are of potential interest in PsA which are not currently approved for the management of PsA. These include anti-B-cell, anti-IL-1, and anti-IL-6 targeted therapies. Notwithstanding the previous demonstration of B-cell aggregates [17] and the upregulation of pro-inflammatory cytokines (including IL-6) in the synovial tissue in active PsA, inhibition of these targets has led to disappointing results [18,135].

The relative lack of efficacy of these agents suggests that the presence of certain cytokines and cells at the site of inflammation does not necessarily indicate their relevance as a therapeutic target in the treatment of the disease. 

## 10. Future Direction and Remaining Questions

The therapies available for treating PsA have evolved markedly over the past decade. Despite the important developments in drug discovery, a considerable research agenda and unanswered questions remain. (Table 2 summarises the key clinical trials evaluating the targeted therapies in PsA)

The need to individualise treatment in this heterogeneous patient group is of paramount importance. In patients with refractory disease, there is often a need to change from one bDMARD to another, and in general, drug survival has been shown to be remarkably short [136,137,138,139]. In addition to the challenge of drug survival, there is no informed strategy for selecting the optimal treatment for an individual patient based on the biology of their disease, as identified by biomarkers. Currently, the most active domain of an individual’s disease (e.g., enthesitis, nail disease, etc.) is used to select a specific target. Furthermore, patients can experience differential responses for their different psoriatic disease manifestations, an example being that therapies targeting the IL-17 pathway can result in dramatic psoriasis responses while improvement in peripheral arthritis may not be as impressive.

Immunophenotyping based upon T-helper cell subsets in the peripheral blood of PsA patients has been put forward as a treatment selection strategy. In a proof-of-principle study, using stratifying of PsA based on a peripheral lymphocyte subtype analysis to inform treatment, there was a signal for improved outcomes [140]. This study represents an exciting potential avenue into precision medicine in PsA with the opportunity to improve our patients’ outcomes. Whether this biological classification and stratification of individual patients should be carried out based on peripheral blood immunophenotyping or synovial tissue profiling and analysis is an important question that remains unanswered.

Most clinical trials assessing novel agents in PsA focus heavily on the efficacy of the therapies to treat peripheral arthritis. As a result, the efficacy relating to the management of other domains (e.g., enthesitis and dactylitis) is generally assessed as secondary endpoints in these trials. This results in challenges for physicians when selecting a therapy to treat these disease domains.

In addition to the lack of biomarkers to inform treatment selection, there is also a paucity of biomarkers to assess disease activity. Acute phase reactants are non-specific and are not always elevated in active disease and are therefore unreliable biological indicators to assess therapeutic responses and disease activity.

If the goal of treatment has not been achieved, whether switching to another immunomodulator or strategies involving the combination or ‘sequential’ use of targeted therapies with different mechanisms of action is another area of research. 

## 11. Conclusions

TNFi was, for a long-time, established as the most effective treatment for all the various disease manifestations of PsA. However, a substantial number of patients do not respond adequately to TNFi. The plethora of new therapies that have emerged from the understanding of the role of the IL-23/IL-17 axis in the pathogenesis of PsA has offered more options for patients with PsA, but at a population level, they have not shown overall improved outcomes when compared with TNFi. Solely long-term data will determine whether these therapies can retard or halt the process of new bone formation, which causes a great deal of pain, deformity, and disability.

Studies are ongoing, and they are hoped to discover further relevant targets, broaden the selection of treatments, and ultimately help optimise patient care. However, for the moment, prevention or cure does not appear to be on the horizon.

## Figures and Tables

**Table 1:** Targeted therapies for psoriatic arthritis.

Therapy	Mechanism of Action
Infliximab	Chimeric monoclonal antibody against TNF-α
Etanercept	Soluble TNF receptor p75-immunoglobulin G1 fusion protein
Adalimumab	Fully human anti-TNF-α monoclonal antibody
Golimumab	Fully human immunoglobulin G1 kappa anti-TNF-α antibody
Certolizumab pegol	Fab fragment of anti-TNF- α monoclonal antibody
Ustekinumab	Fully human immunoglobulin G1 kappa monoclonal antibody against the shared p40 subunit of IL-12 and IL-23
Guselkumab	Fully human immunoglobulin G1 lambda monoclonal antibody against the p19 subunit of IL-23
Risankizumab	Fully human immunoglobulin G1 lambda monoclonal antibody against the p19 subunit of IL-23
Secukinumab	Fully human immunoglobulin G1 kappa monoclonal antibody against IL-17A
Ixekizumab	Humanised immunoglobulin G4 monoclonal antibody against IL-17A
Brodalumab	Fully human immunoglobulin G2 monoclonal antibody against IL-17RA
Bimekizumab	Humanised immunoglobulin G1 monoclonal antibody against IL-17A and IL-17F
Tofacitinib	Inhibitor of JAK1 and JAK3
Upadacitinib	Selective inhibitor of JAK1
Filgotinib	Selective inhibitor of JAK1
Deucravacitinib	Selective TYK2 inhibitor
Abatacept	Selective T cell co-stimulator inhibitor

**Table 2:** Clinical trials in psoriatic arthritis.

Study	Comparison	Population	TNFi-IR (%)	MTX Use (%)	Primary Outcome	Primary Endpoint (Weeks)	ACR20 (%)
TNF-α inhibitors							
Antoni et al. (IMPACT 2) (N = 200) [36]	IFX vs. PBO	csDMARD-IR or NSAID-IR	0	46	ACR20	14	58 (IFX) vs. 11 (PBO) *
Mease et al. (N = 205) [35]	ETN vs. PBO	NSAID-IR	0	41	ACR20	12	59 (ETN) vs. 15 (PBO) *
Mease et al. (ADEPT) (N = 313) [34]	ADA vs. PBO	NSAID-IR	0	50.5	ACR20	12	58(ADA) vs. 14 (PBO) *
Kavanaugh et al. (GO-REVEAL) (N = 405) [37]	GOL (50 mg, 100 mg) vs. PBO	NSAID-IR	0	48	ACR20	24	48 (combined) vs. 9 (PBO) *
Mease et al. (RAPID-PsA) (N = 409) [38]	CTZ (200 mg Q2W, 400 mg Q4W) vs. PBO	csDMARD-IR	NR 38.9 prior TNFi	64	ACR20	12	58 (Q2W) 51.9 (Q4W) vs. 24.3 (PBO) *
IL-12, 23p40 inhibitor							
McInnes et al. (PSUMMIT 1) (N = 615) [55]	UST (45 mg, 90 mg) vs. PBO	csDMARD-IR	0	48	ACR20	24	42.4 (45 mg) 49.5 (90 mg) 22.8 (PBO) *
Ritchlin et al. (PSUMMIT 2) (N = 312) [56]	UST (45 mg, 90 mg) vs. PBO	Mixed csDMARD-IR/TNFi-IR	57.7	50	ACR20	24	43.8 (combined) 20.2 (PBO) *
Il-23p19 inhibitors							
Deodhar et al. (DISCOVER-1) (N = 381) [67]	GKM (Q4W, Q8W) vs. PBO	Mixed csDMARD-IR/Apremilast-IR/TNFi-IR	12	55	ACR20	24	59 (Q4W) 52 (Q8W) 22 (PBO) *
Mease et al. (DISCOVER-2) (N = 739) [68]	GKM (Q4W, Q8W) vs. PBO	csDMARD-IR	0	60	ACR20	24	64 (Q4W) 64 (Q8W) 33 (PBO) *
Coates et al. (COSMOS) (N = 285) [73]	GKM vs. PBO	TNFi-IR	84	55	ACR20	24	44.4 (GKM) vs. 19.8 (PBO) *
Kristensen et al. (KEEPsAKE-1) (N = 964) [141]	RKM vs. PBO	csDMARD-IR	0	65.2	ACR20	24	57.3 (RKM) vs. 33.5 (PBO) *
Östör et al. (KEEPsAKE-2) (N = 443) [142]	RKM vs. PBO	Mixed csDMARD-IR/Bio-IR	46	47.1	ACR20	24	51.3 (RKM) vs. 26.5 (PBO) *
Il-17A inhibitors							
Mease et al. (FUTURE 1) (N = 606) [88]	SEC (150 mg, 75 mg) vs. PBO	Mixed csDMARD-IR/TNFi-IR	29.4	61	ACR20	24	50 (150 mg) 50.5 (75 mg) 17.3 (PBO)
McInnes et al. (FUTURE 2) (N = 397) [89]	SEC (300 mg, 150 mg, 75 mg) vs. PBO	Mixed csDMARD-IR/TNFi-IR	35	47	ACR20	24	54 (300 mg) 51 (150 mg) 29 (75 mg) 15 (PBO) *
McInnes et al. (EXCEED) (N = 853) [107]	SEC vs. ADA	csDMARD-IR	0	85	ACR20	52	67 (SEC) 62 (ADA)
Mease et al. (SPIRIT-P1) (N = 417) [103]	IXE (Q2W, Q4W) vs. PBO vs. ADA (reference arm)	csDMARD-IR	0	54.2	ACR20	24	62.1 (Q2W) 57.9 (Q4W) 30.2 (PBO) * 57.4 (ADA)
Nash et al. (SPIRIT-P2) (N = 363) [102]	IXE (Q2W, Q4W) vs. PBO	TNFi-IR	91	41	ACR20	24	48 (Q2W) 53 (Q4W) 20 (PBO) *
IL-17RA inhibitor							
Mease et al. (AMVISION-1 and AMVISION-2) (N = 962) [108]	Brodalumab (140 mg, 210 mg) vs. PBO	Mixed csDMARD-IR/Bio-IR	NR	NR	ACR20	16	45.8 (140 mg) 47.9 (210 mg) 20.9 (PBO) *
IL-17A/F inhibitor							
McInnes et al. (BE-OPTIMAL) (N = 852) [113]	BKM vs. PBO vs. ADA (reference arm)	csDMARD-IR	0	58	ACR50 (44% BKZ vs. 10% PBO) *	16	62.2 (BKZ) vs. 24 (PBO) * 68.6 (ADA)
Merola et al. (BE-COMPLETE) (N = 400) [114]	BKM vs. PBO	TNFi-IR	88	43	ACR50 (43% BKZ vs. 7% PBO) *	16	67 (BKZ) vs. 15.8 (PBO) *
JAK inhibitors							
Mease et al. (OPAL Broaden) (N = 422) [121]	TOFA (5 mg BD, 10 mg BD) vs. PBO vs. ADA (reference arm)	csDMARD-IR	0	84	ACR20	12	50 (5 mg) vs. 61 (10 mg) vs. 33 (PBO) * 52% (ADA)
Gladman et al. (OPAL Beyond) (N = 394) [122]	TOFA (5 mg BD, 10 mg BD) vs. PBO	TNFi-IR	100	73	ACR20	12	50 (5 mg) vs. 47 (10 mg) vs. 24 (PBO) *
McInnes et al. (SELECT-PsA 1) (N = 1704) [126]	UPA (15 mg, 30 mg) vs. PBO vs. ADA (reference arm)	csDMARD-IR	0	84	ACR20	12	70.6 (15 mg) vs. 78.5 (30 mg) vs. 36.2 (PBO) * 65 (ADA)
Mease et al. (SELECT-PSA 2) (N = 641) [127]	UPA (15 mg, 30 mg) vs. PBO	Bio-IR	NR 92% Bio-IR	37	ACR20	12	56.9 (15 mg) vs. 63.8 (30 mg) vs. 24.1 (PBO) *
Mease et al. (EQUATOR) (N = 131) [128]	Filgotinib vs. PBO	csDMARD-IR	0	55	ACR20	16	80 (filgotinib) vs. 33 (PBO) *
Other targets							
Mease et al. (N = 203) [133]	Deucravacitinib (6 mg, 12 mg) vs. PBO	Mixed csDMARD-IR/TNFi-IR	NR 15.8% prior TNFi	54.7	ACR20	16	52.9 (6 mg) vs. 62.7 (12 mg) vs. 31.8 (PBO) *
Mease et al. (ASTRAEA) (N = 424) [134]	ABA vs. PBO	Mixed csDMARD-IR/TNFi-IR	61	60	ACR20	24	39.4 (ABA) vs. 22.3 (PBO) *

* Statistically significant result; MTX, methotrexate; TNFi-IR, tumour necrosis factor inhibitor inadequate response; csDMARD-IR, conventional synthetic disease modifying anti-rheumatic drug inadequate response; Bio-IR, biologic inadequate response; NSAID-IR, non-steroidal anti-inflammatory drug inadequate response; IFX, infliximab; PBO, placebo; ETN, etanercept; ADA, adalimumab; GOL, golimumab; CTZ, certolizumab pegol; UST, ustekinumab; GKM, guselkumab; RKM, rizankisumab; SEC, secukinumab; IXE, ixekizumab; BKM, bimekizumab; TOFA, tofacitinib; UPA, upadacitinib; ABA, abatacept.

## Data Availability

Not applicable.
